# Spatial localization of electromyographic amplitude distributions associated to the activation of dorsal forearm muscles

**DOI:** 10.3389/fphys.2013.00367

**Published:** 2013-12-13

**Authors:** Alessio Gallina, Alberto Botter

**Affiliations:** Laboratory for Engineering of the Neuromuscular System (LISiN), Dipartimento di Elettronica e Telecomunicazioni, Politecnico di TorinoTorino, Italy

**Keywords:** forearm, wrist, finger, muscle compartmentalization, electromyography, high-density surface EMG

## Abstract

In this study we investigated whether the spatial distribution of surface electromyographic (EMG) amplitude can be used to describe the activation of muscle portions with different biomechanical actions. Ten healthy subjects performed isometric contractions aimed to selectively activate a number of forearm muscles or muscle subportions. Monopolar electromyographic signals were collected with an electrode grid of 128 electrodes placed on the proximal, dorsal portion of the forearm. The monopolar EMG amplitude [root mean square (RMS) value] distribution was calculated for each contraction, and high-amplitude channels were identified through an automatic procedure; the position of the EMG source was estimated with the barycenter of these channels. Each of the contractions tested was associated to a specific EMG amplitude distribution, whose location in space was consistent with the expected anatomical position of the main agonist muscle (or subportion). The position of each source was significantly different from the others in at least one direction (ANOVA; transversally to the forearm: *P* < 0.01, *F* = 125.92; longitudinally: *P* < 0.01, *F* = 35.83). With such an approach, we could distinguish the spatial position of EMG distributions related to the activation of contiguous muscles [e.g., extensor carpi ulnaris (ECU) and extensor digitorum communis (EDC)], different heads of the same muscle (i.e., extensor carpi radialis (ECR) brevis and longus) and different functional compartments (i.e., EDC, middle, and ring fingers). These findings are discussed in terms of how forces along a given direction can be produced by recruiting population of motor units clustered not only in specific muscles, but also in muscle sub-portions. In addition, this study supports the use of high-density EMG systems to characterize the activation of muscle subportions with different biomechanical actions.

## Introduction

Daily activities require the availability of multiple degrees of freedom in the upper limb joints. In order to provide the wrist and the hand with such a variety of movements, muscles with different lines of action origin from the elbow region and run through the forearm inserting at the hand level. Each of these muscles is highly specialized, its function in terms of direction of the movement being defined by its architectural features (Lieber et al., 1990). Besides, compartmentalization has been described in most of the forearm muscles (Segal et al., 2002). For instance, it is widely known that fingers can be selectively extended with a certain degree of independence (Van Duinen et al., 2009), an that this is possibly related to “task group” motor units with fibers clustered in limited portions of the EDC muscle (EDC, Riek and Bawa, 1992). Similarly, although considered as a muscle with a single function in some studies (Finsen et al., 2005; Mananas et al., 2005; Alizadehkhaiyat et al., 2007; Rojas et al., 2007), the two heads of the extensor carpi radialis muscle (ECR) produce force and movement in different directions (i.e., ECR brevis: predominantly wrist extension; ECR longus: predominantly radial deviation, Bawa et al., 2000; Sagae et al., 2010). Interestingly, it was also proven that either head can be activated with a certain degree of independence with respect to the other (Riek et al., 2000). Anatomical partitioning was also described within the extensor carpi ulnaris muscle (ECU), with up to four partitions defined in virtue of nerve branches insertion and muscle fiber orientation (Segal et al., 2002). As all these muscles (or muscle compartments) responsible for different wrist and finger movements are placed in a relatively small body area (the dorsal portion of the forearm), the possibility to discriminate their activation is essential for studies investigating wrist and hand motor control.

Electromyographic (EMG) technique is the gold standard for the analysis of muscle activation. The classical bipolar montage was found to be inadequate for the assessment of single forearm muscles (Finsen et al., 2005). One of the major causes accounted for this poor selectivity of large, surface electrodes is the high level of crosstalk (i.e., EMG signals generated by muscles different from the target one, De Luca and Merletti, 1988) that can be observed between signals recorded in multiple sites of the proximal forearm (Mogk and Keir, 2003). This issue might be limited with a careful positioning of electrode pairs (Leijnse et al., 2008a) with small inter-electrode distance (Hoffman and Strick, 1999). However, these solutions might present issues related to a number of factors, e.g., (i) inter-subject variation of muscle architecture and compartmentalization may affect the estimation of EMG activity if the electrode position is defined on the basis of anatomical references; (ii) muscle activity might not be appropriately represented if the pick-up volume of the bipolar electrodes is not big enough to collect EMG signals from a representative amount of the active motor units; (iii) detection systems with small inter-electrode distance need an accurate placement, thus the possibility to collect signals influenced by anatomical factors such as thickness of the interposed tissue and position of the innervation zone is increased. Altogether these factors might affect the EMG recordings, potentially limiting the amount of physiological information that can extracted from the signals collected.

Another approach for increasing the accuracy of muscle activity description is to characterize the distribution of electrical activity of the action potentials on the skin; indeed, it has been proven that by using high-density EMG systems (i.e., groups of electrodes systematically organized in arrays or grids) it is possible to define where the electrical activity of recruited motor units is best represented (Roeleveld et al., 1997), thus differentiating the EMG signal produced from a source under the recording electrodes from that of motor units localized farther away. For instance, by taking advantage of bi-dimensional electrode grids, the spatial distribution of EMG activity over the forearm was used to discriminate the activity of ECR and EDC in response to voluntary and elicited contractions (Van Elswijk et al., 2008). In addition, the spatial distribution of EMG activity on the arm and forearm was used to discriminate different contractions of elbow flexion/extension and forearm pronation/supination (Rojas-Martínez et al., 2013), also resulting in estimates of EMG amplitude more reliable than classical, bipolar configuration (Rojas-Martínez et al., 2012). Whether the spatial features of the EMG amplitude distribution on the forearm can be useful to distinguish the activation of muscles (and muscle compartments) with different biomechanical action is still unknown.

The aim of this work was to investigate whether it is possible to spatially localize over the forearm the EMG distributions associated to different motor tasks. The tasks tested were isometric contractions aimed to selectively activate muscles localized in the dorsal, proximal portion of the forearm (ECR, ECU, EDC, extensor digiti minimi, brachioradialis). As the EMG amplitude is highest for the electrodes above the active motor units, we hypothesize that: (i) each contraction is associated to a specific EMG amplitude distribution; (ii) this distribution is spatially localized on the skin above the main agonist muscle (subportion) activated during the task; (iii) muscles (or muscle subportions) with different biomechanical actions can be discriminated and localized on the basis of the spatial features of the EMG amplitude distribution. Information about which location of the forearm yields the highest EMG activity for each contraction direction, as well as if the EMG activity of pairs of contractions are more easily distinguished along either the longitudinal or the transverse forearm axis, is provided in this study.

## Methods

### Subjects

Ten healthy subjects were tested (six males and four females, age: 27 ± 5 years, height: 173 ± 9 cm, weight: 67 ± 11 kg). All subjects were pain-free at the time of the experiment and reported no known upper limb pathologies. An informed consent was signed by each subject before the beginning of the experiment. All the tests were approved by the local Ethic Committee.

### Protocol

Before the beginning of the experimental session, the subjects were allowed to familiarize with the experimental setup and with the contractions required for the experimental procedure. After a short warm up, maximal voluntary contractions of wrist extensors and ulnar/radial deviators were measured with three separate contractions for each direction. Force measures were performed using an isometric force brace with a load cell (full scale: 200 N) positioned above the 3rd metacarpal bone during extensions and laterally to the head of the second and the fifth metacarpal bones during deviations. For all the contractions, the subject was asked to keep the hand as a fist, without clenching his/her fingers. In order to limit possible compensations using forces around the other joints (elbow and shoulder), the upper limb was fixed with the shoulder at 90° in anterior flexion with the elbow fully extended. The forearm was pronated, in order to limit the contribution of the ECU during wrist extension (Sagae et al., 2010). The tested contractions were (in random order): wrist extension, radial deviation and ulnar deviation, at 20, 50, and 80% of the maximal voluntary contraction, elbow flexion against manual resistance (asking the subject to exert a low-force contraction, about 20% of their maximal voluntary contraction) and metacarpo-phalangeal joint extension, lifting the middle, ring or little finger as much as possible without moving the others. Contractions at 20 and 50% MVC were 10 s long, whereas all the others were 5 s long. For wrist extension, radial and ulnar deviation, the contractions were produced at three different force levels in order to describe possible changes in the distribution of the surface EMG amplitude related to preferential activation of muscle subportions at different force levels. Index finger and thumb extension were not taken into account, as the muscles responsible for those movements are positioned distally with respect to the forearm portion covered by the electrode grid.

### Data acquisition

**Figure 1:**
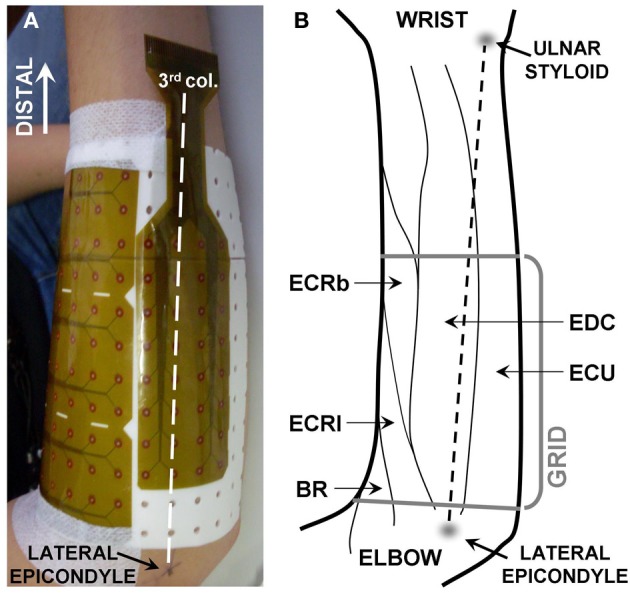
**(A)** Position of the detection system. The third column of electrodes (being the first on the ulnar side) was aligned with the line linking the lateral epicondyle with the ulnar styloid. The grid consisted of 128 electrodes organized in 12 columns by 12 rows, with two groups of 4 × 2 electrodes missing in the ulnar lateral corners. The subject's hand was kept in pronation. **(B)** Anatomy of the muscles of the proximal, dorsal forearm (drawing based on anatomical indications from Leijnse et al., 2008b). BR, brachioradialis; ECRl, extensor carpi radialis longus; ECRb, extensor carpi radialis brevis; EDC, extensor digitorum communis; ECU, extensors carpi ulnaris.

**Figure 2:**
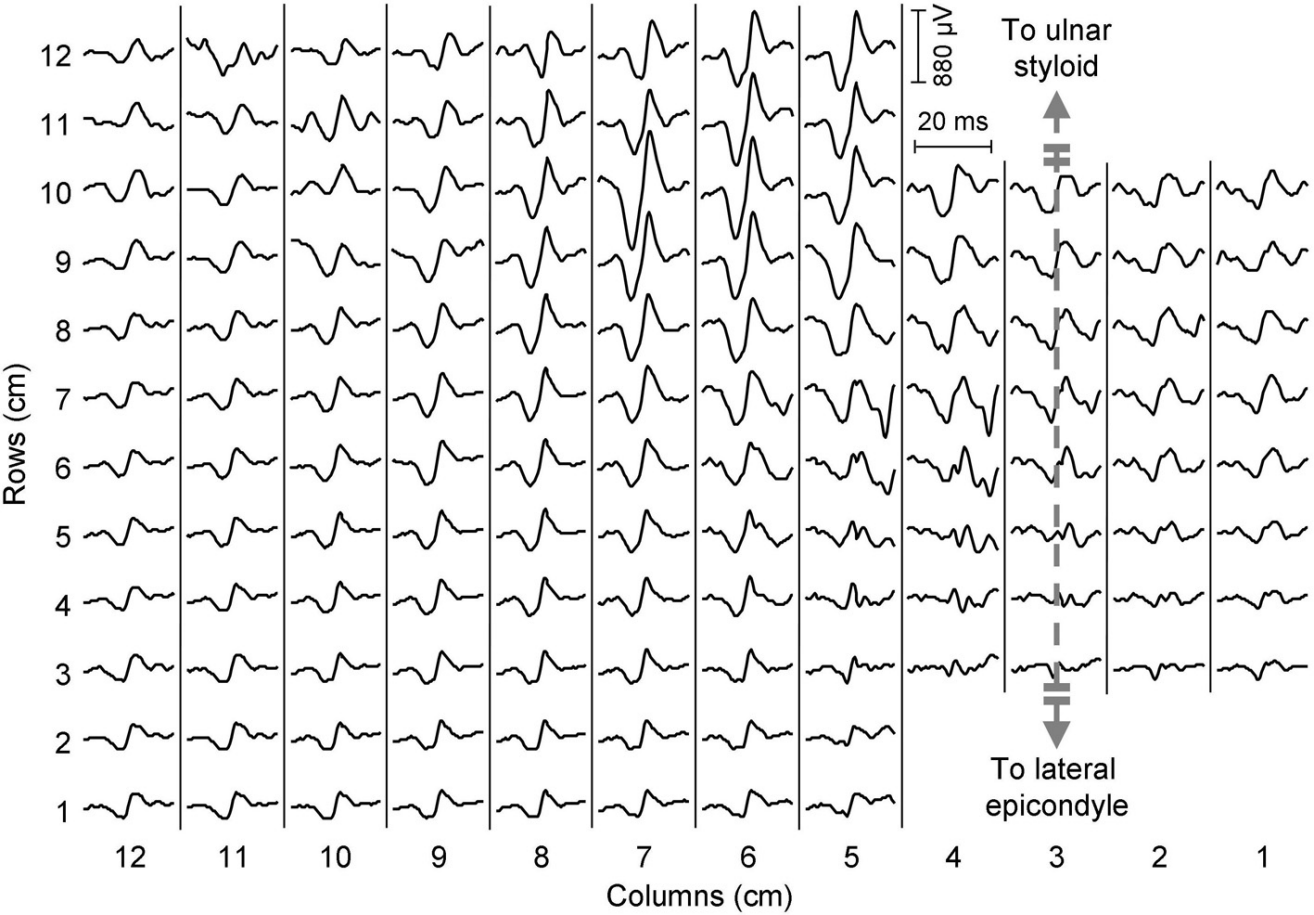
**Epoch of EMG signals from a representative subject during a 20% MVC contraction (wrist extension).** A motor unit action potential centered in the distal portion of the grid can be observed.

Before placing the electrode grid, the length (lateral epicondyle—ulnar styloid) and the proximal circumference of the forearm (2 cm distal to radial head) were measured, and a line between the lateral epicondyle and the ulnar styloid was drawn. A custom-made grid of 128 electrodes for EMG detection, organized in 12 rows by 8 columns (radial side) plus 8 rows by 4 columns (ulnar side, see Figure 1A for details) was placed on the right forearm of each subject. The distance between consecutive electrodes was 10 mm. The third column of the grid was aligned with the epicondyle-styloid line (Figure 1A), and the proximal edge of the grid was placed as close as possible to the elbow joint (5 mm distal to the radial head). Before applying the electrode grid, muscle position was approximately defined by palpation during active contractions. This electrode configuration, which was determined with pilot tests, allowed to collect EMG activity from all the muscles located in the dorsal, proximal portion of the forearm. The choice of the detection system (density of the grid and number of electrodes) was limited by the number of channels of the amplifier and by size and interelectrode distance of electrode grids currently available. For this reason, the representation of muscles close to the edges of the grid (ECU, brachioradialis) might be partial in subjects with large forearm circumference; it is also likely that only a small portion of the extensor digiti minimi was covered by the electrodes. An outline of the forearm muscle anatomy is provided in Figure 1B. EMG signals were acquired in monopolar configuration and amplified 200–5000 times and then digitized at 2048 samples/s using a 12 bit A/D converter (EMG-USB amplifier, OT-Bioelettronica, Italy). Force signals were amplified (MISO 2, OT-Bioelettronica, Italy) and acquired simultaneously with EMG signals through the auxiliary output of the EMG amplifier. Before the data processing, EMG signals were band-pass filtered (10–400 Hz). Signal quality was assessed through visual analysis of the raw EMG signals both in time and in frequency domain. Isolated bad channels due to bad skin-electrode contact were replaced using a linear interpolation of the neighboring channels. The median number of bad channels was 4 out of 128 (interquartile interval: 3–6). An example of monopolar EMG signals is shown in Figure 2.

### Data processing

**Figure 3:**
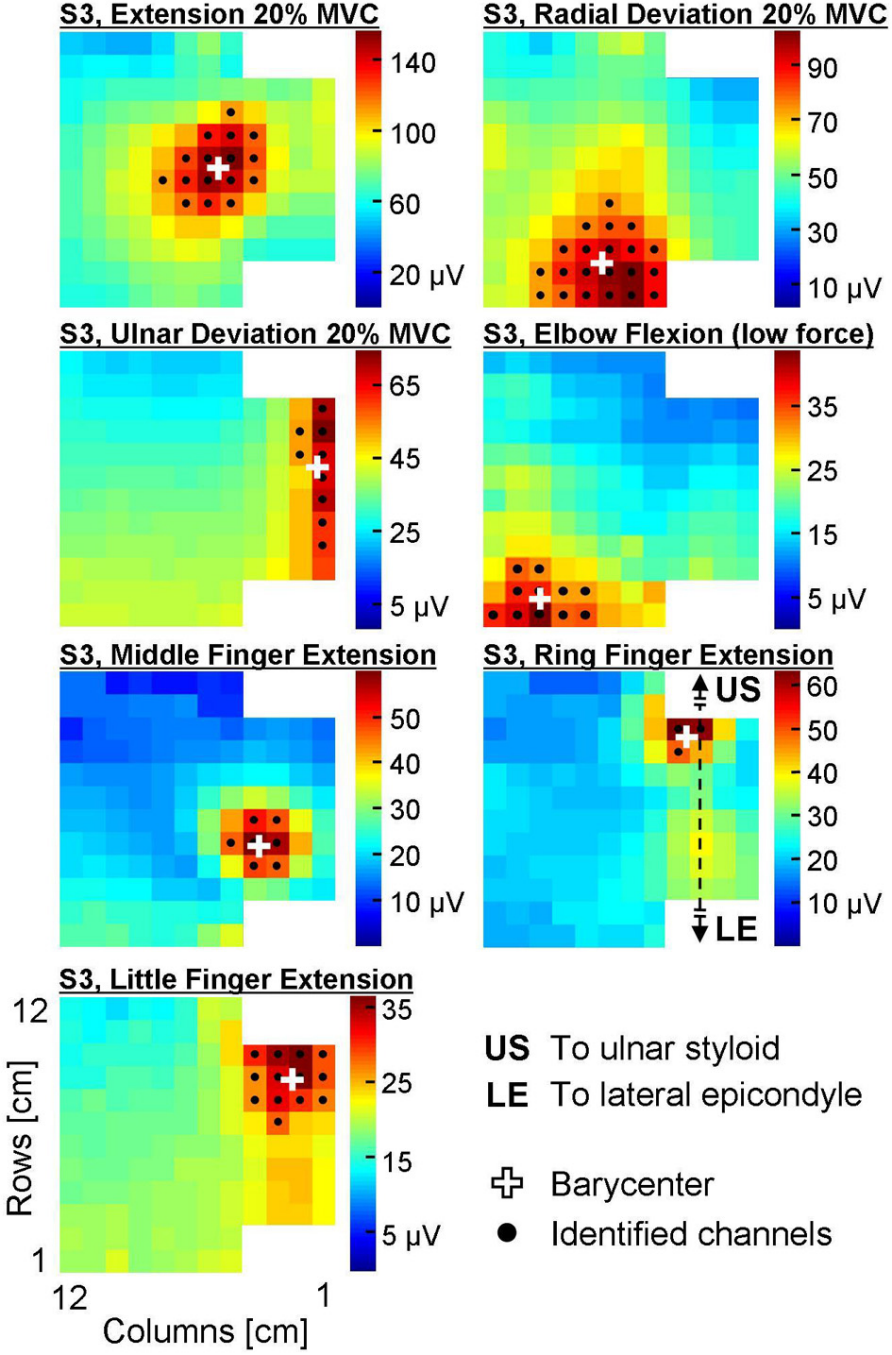
**Surface EMG amplitude distribution (RMS) over the skin during seven different contractions of a representative subject (S3).** The colorbar of each map ranges between 0 and the peak amplitude of the map. Black dots identify the channels automatically identified with the segmentation algorithm, white crosses are the barycenters of these channels.

For wrist extension/deviation contractions, a 3 s epoch corresponding to the steadiest force (assessed computing the standard deviation of the force signal over a 3 s moving window) were extracted and analyzed. For finger extension and elbow flexion tasks, the 3 s epoch in the central portion of the contraction was considered. The monopolar amplitude distribution of EMG over the forearm was obtained computing the root mean square (RMS) of each channel of the grid during the selected epoch. An automatic algorithm was applied to the monopolar amplitude distribution to localize which channels of the electrode grid recorded a relevantly high EMG activity (Vieira et al., 2010) and therefore identify the location of the active regions. This algorithm applies a watershed segmentation to the equalized EMG amplitude map to identify clusters with different neuromuscular activity; afterwards, the channels within each cluster whose amplitude is higher than 70% of the maximal value are considered as the relevant channels. In this study, when more than one cluster was identified in the first stage of the algorithm, only the cluster with the highest EMG peak amplitude was considered. The position of the active area (proximal-distal and medio-lateral coordinates of the barycenter of the relevant channels) was extracted and normalized with respect to the anatomical measures of the subjects (length and proximal circumference of the forearm). Lastly, the barycenter of each group of channels within the selected cluster was computed and used to define the position of the active area and eventually to characterize variations of the amplitude distribution between conditions (Van Elswijk et al., 2008). An example of EMG amplitude distributions and of their segmentation is provided in Figure 3.

### Statistical analysis

All the statistical analyses were performed using the software Sigmaplot 12. The assumptions of normal distribution were respected for all data sets (Shapiro-Wilk test). The possibility to distinguish the position of different muscles was analyzed using an Two-Way analysis of variance (ANOVA), testing the effects of the factors “subject” and “direction of force” separately on the X- and on the Y- coordinate of the barycenter of the amplitude distribution. For wrist extension, radial and ulnar deviations, only the contraction performed at the lowest force level (20% MVC) was considered. In order to test whether the position of the active muscle area changes with the level of force exerted, the effect of force level, force direction and subject on the X- and Y- barycenter of the amplitude distribution was investigated with a Three-Way ANOVA. *Post-hoc* tests were performed using the Holm-Sidak test. Significance was set at *P* < 0.05. Data are reported as mean and standard deviation.

## Results

The average forearm length was 25.2 ± 1.8 cm (mean ± standard deviation), whereas the forearm circumference in its proximal portion was 25.5 ± 2.2 cm. An example of EMG amplitude distributions and of their segmentation is provided in Figure 3.

### Effect of the task performed on the EMG amplitude distribution

**Figure 4:**
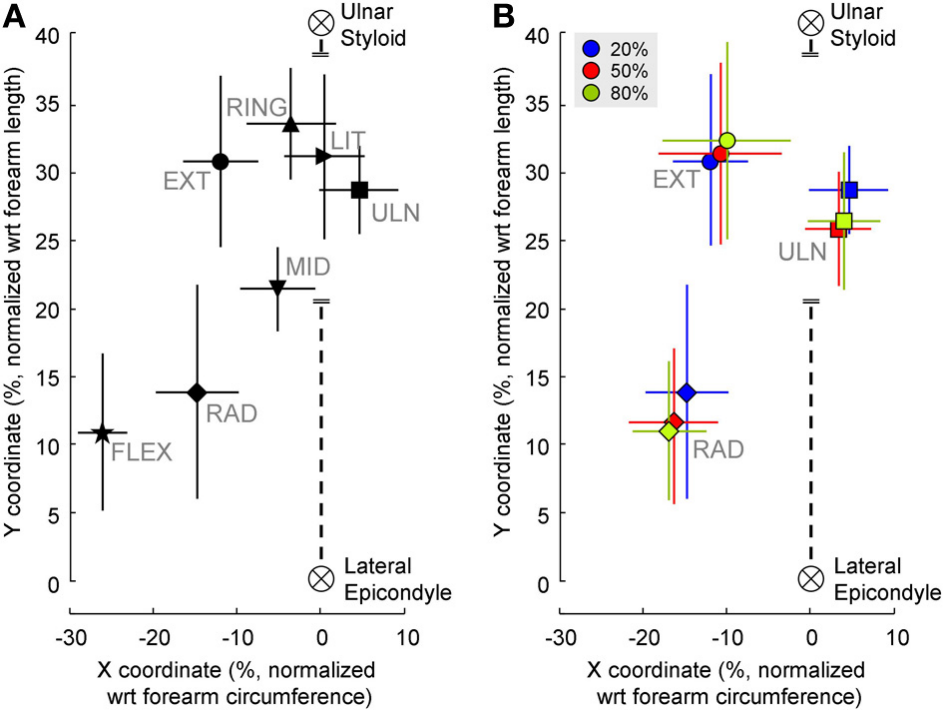
**(A)** Position of the clusters' barycenter identified from EMG amplitude distributions during the contractions tested. For wrist extension, radial and ulnar deviation only the position of the barycenter extracted from the 20% MVC contraction is shown. **(B)** Position of the clusters' barycenter identified from EMG amplitude distributions during wrist extension and radial deviation contractions at different force levels. The black, dashed line represents the third column of electrodes. The coordinates of each subject were normalized with respect to the forearm circumference (X-axis) and forearm length (Y-axis). Symbols represent the mean values of X and Y coordinates of all subjects pooled together, vertical and horizontal lines are the standard deviation.

Figure 4A shows the barycenter position for different contractions. The position of the active cluster during different contractions could be always be distinguished along either the proximal/distal (*F* = 35.83, *P* < 0.01) or the medio-lateral direction (*F* = 125.92, *P* < 0.01); most contractions could be distinguished along both axes. Table 1 shows whether the position of the active areas of each pair of contractions can be distinguished on the medio-lateral (X) and proximal-distal (Y) direction (Holm-Sidak *post-hoc* test). When the X coordinate was considered, it was possible to discriminate all pairs of contractions except wrist extension vs. radial deviation and middle vs. ring finger extension. Along the forearm (Y coordinate), instead, it was not possible to distinguish (i) wrist extension from ulnar deviation, ring and little finger extension; (ii) radial deviation from elbow flexion; (iii) ulnar deviation from ring and little finger extension, and (iv) ring from little finger extension. The factor subject influenced the medio-lateral (*F* = 4.49, *P* < 0.01) but not the proximal-distal (*F* = 0.93, *P* = 0.5) position of the cluster.

**Table 1:**
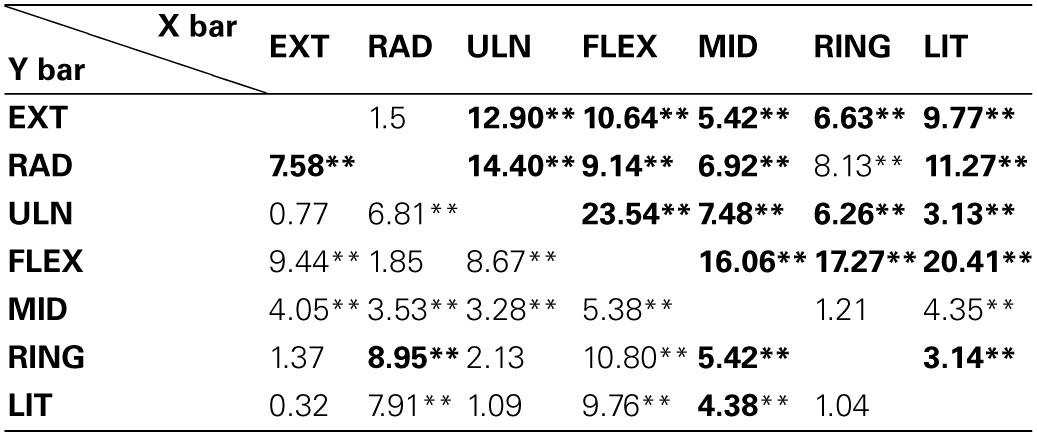
**Comparison of the barycenter position among contractions**.

Results of post-hoc comparisons (Holm-Sidak t-values) are shown for medio-lateral (X, top-right half of the table indicated with X bar) and proximal-distal coordinates of the barycenter (Y, bottom-left half of the table indicated with Y bar). Bold highlights which coordinate best discriminate each pair of contractions. For example, EMG amplitude distributions associated to wrist radial (RAD) and ulnar (ULN) deviations can be distinguished with p < 0.01 (^**^) in both directions, however, the Holm-Sidak t-value is higher in the X (14.4, top-right half of the table) than in the Y axis (6.81, bottom-left half of the table), indicating that the amplitude distributions can be better distinguished transversally to the forearm. EXT, Wrist extension; RAD, Wrist radial deviation; ULN, Wrist ulnar deviation; FLEX, Elbow flexion; MID, Extension of the middle finger; RING, Extension of the ring finger; LIT, Extension of the little finger. ^**^, p < 0.01.

### Effect of contraction intensity on the EMG amplitude distribution

Figure 4B shows the effect of different force levels on the barycenter position. When higher-force contractions were considered (for extension, radial and ulnar deviation), the position of the normalized barycenter was influenced by both subject (medio-lateral: *F* = 10.12, *P* < 0.01; proximal-distal: *F* = 2.32, *P* = 0.03) and force direction (medio-lateral: *F* = 239.8, *P* < 0.01; proximal-distal: *F* = 233.78, *P* < 0.01). Instead, no effects of force level on either axes was found (medio-lateral: *F* = 0.82, *P* = 0.45; proximal-distal: *F* = 2.16, *P* = 0.13).

## Discussion

In this study, we analyzed the surface EMG amplitude distribution collected during contractions aimed at selectively activating a number of dorsal forearm muscles. Each contraction corresponded to a specific EMG distribution with relevantly high amplitude values in few channels only, localized above the targeted, main agonist. We demonstrated that the spatial properties of the monopolar EMG amplitude distribution over the proximal forearm can be used to discriminate different contractions. In addition, we showed that the coordinates of the active area were not influenced by the level of force exerted in isometric wrist contractions.

### Localization of the EMG sources

For each selective contraction, the barycenter of the channels identified from monopolar EMG amplitude maps agrees with the position of the main agonists (Figures 3, 4) as described in the literature (Leijnse et al., 2008b). Specifically, the area showing the highest EMG activity did not correspond to the whole muscle size, but rather to a small portion of its longitudinal dimension. For instance, the EDC compartment whose action is the extension of the metacarpo-phalangeal joint of the middle finger was reported to extend along about 50% of the forearm length origin (from −4.0 to 47.3% of the radius length, 0% being the radio-ulnar joint, Leijnse et al., 2008b). In this experiment, we localized a relevantly high EMG activity of middle finger extensors in a portion of the grid not longer than 3 cm (about 12% of the average forearm length) along the proximal-distal direction (Figure 3). This portion of the muscle corresponds to its main innervation zone, which is known to be the portion of the muscle showing the highest amplitude of monopolar EMG signals (Kleine et al., 2000). Therefore, monopolar amplitude distribution shows a single peak above this region, with signals progressively lower along the muscle fiber; by contrast, differential signals show high amplitude along the whole muscle fiber and a sudden dip above the innervation zone (Kleine et al., 2000; Rodriguez-Falces et al., 2013). Thus, information on the spatial distribution of EMG amplitude might be more easily extracted from monopolar rather than single differential signals in virtue of the single, localized peak of the monopolars. Remarkably, also in other studies described in the literature this detection modality was preferred (Van Elswijk et al., 2008; Rojas-Martínez et al., 2012, , Alonso and Merletti). Further reasons supporting the use of monopolar signals rather than single differentials for the purposes of this study are related to the fact that many muscles with different architectures and fiber orientation are present in few square centimeters. This implies that: (i) the RMS amplitude calculated on monopolar signals might be more representative of muscle activity as it is not dependent on the direction of the muscle fibers with respect to the electrodes; (ii) due to the presence of many muscles in the forearm, spatial filters might be calculated from monopolar signals generated by different muscles.

### Discrimination of different contractions

As shown in Table 1, most EMG amplitude distributions could be discriminated from the others considering both the proximal-distal and the medio-lateral coordinates of the barycenter of the identified cluster. Most pairs of contractions (17 out of 21, Table 1) were distinguished with higher statistical significance in the transversal than in the longitudinal axis; some contractions, such as extension/radial deviation and ring/middle finger extension, could be separated only considering the proximal-distal rather than the medio-lateral coordinates of the barycenter. This is related to the anatomy of the muscles responsible for those contractions; for example, the possibility to differentiate extension from radial deviation contractions along the proximal-distal direction is in line with the suggestion of Riek et al. (2000) to insert needles at different percentage of the forearm length to obtain a selective recording from the ECR longus (proximal, wrist radial deviator) and ECR brevis (distal, wrist extension). The relatively large distance between the centers of these two EMG sources (18% of the forearm length, about 4.5 cm considering an average forearm length of 25.2 cm) might also partially explain why studies using intramuscular detection systems could not identify task-group motor units in the ECR (Riek and Bawa, 1992). Besides, our results confirm that studies using intramuscular recordings to assess the activity of the ECR might not represent the activity of the whole muscle, but rather of the muscle head in which needles or wires are inserted (Riek et al., 2000). Indeed, the ECR was shown not to contribute to radial deviation forces when its activation was assessed with a needle inserted in the bulky part of the ECR, roughly corresponding to the ECR brevis (Finsen et al., 2005), whose main function is wrist extension. Concerning the EDC, instead, a partial independence of motor unit pools extending different fingers has previously been described in the literature (Van Duinen et al., 2009); specifically, two subpopulations of EDC motor units partially overlapping in space control the extension of middle and ring fingers (Riek and Bawa, 1992). In this study we observed significant differences in the spatial localization of the EMG amplitude distribution associated to the extension of each of the two fingers; this suggests that subjects performed the tasks mainly by recruiting task-specific groups of motor units clustered in specific portions of the EDC. We believe that such a selective activation of the EDC functional compartments could be observed because of the well controlled conditions imposed in this experiment, i.e., (i) the contraction was a simple, isometric task; (ii) the amount of finger lifting was self-selected at a comfortable height; (iii) subjects were specifically instructed to pay attention not to lift the other fingers; (iv) no feedback that could distract the subject, such as a force profile to match or a timing for finger tapping, was provided. It should be noted that our finding does not challenge the notion that muscle fibers localized in the two compartments cannot be controlled in a completely independent manner (Riek and Bawa, 1992; Van Duinen et al., 2009). Indeed, in the panel of Figure 2 referring to ring finger extension, two amplitude peaks can be observed: a high-amplitude one localized close to the average position of the EMG amplitude distribution identified during ring finger extensions, and a second, lower one referable to middle finger extension. Since the analysis we performed allowed to identify the channels with the highest EMG amplitude disregarding other low-amplitude areas, no inferences about the activation of other compartments can be done.

### Effects of contraction intensity on the EMG amplitude distribution

Within each of the contractions tested at different percentages of MVC, the position of the active area was not significantly influenced by the level of force exerted (Figure 4B). This finding supports our estimate of the spatial localization of the EMG source, as no significant shifts of the sources were observed across the range of the different force levels tested. In addition, our results suggest that variation of the within-muscle distribution of activity as a function of the force exerted are minimal, not equally represented among subjects or smaller than the spatial resolution of our detection system (10 mm). It should be noticed that this analysis did not evaluate the load sharing, i.e., whether the relative activation of synergist muscles varies with the level of force exerted; indeed, in the current analysis relative changes of the EMG amplitude in clusters other than the main one do not influence the position of the main active area, and therefore of its barycenter.

### Inter-subject variability of the EMG amplitude distribution

A main effect of the subject on the medio-lateral coordinate of the barycenter was observed when different contractions were compared; a significant effect of the subject on both medio-lateral and proximal-distal coordinates of the barycenter of the cluster was also reported when different force levels were compared. There are many factors that can account for this inter-subject variability. Among the methodological factors, differences related to errors in the positioning of the grid and to the size of the grid with respect to subjects' forearm might result in imprecise localization of the EMG amplitude distributions with respect to the forearm anatomical reference. We attempted to limit the influence of these factors by placing the grid according to anatomical landmarks easily identifiable (radial head and ulnar styloid); besides, the coordinates were normalized with respect to each subject's forearm circumference and length. A part of this variability might also be explained by physiological factors, such as the use of accessory muscles during finger tasks (e.g., little finger extension performed with either a EDC compartment or the extensor digiti minimi muscle, Leijnse et al., 2008a), the non-complete selectivity of the contractions required to isolate contractions of single muscles as documented by intramuscular recordings (Riek et al., 2000) and the use of a synergist muscle for fixation purposes (Finsen et al., 2005). A possible, further explanation for this inter-subject variability might be the existence of both anatomical and functional muscle compartments, largely documented within forearm muscles (Segal et al., 2002). For example, anatomical studies on the ECR brevis documented a large variability in the intra-muscular pattern of muscle innervation, possibly related to muscle compartmentalization (Ravichandiran et al., 2012). Different roles of the proximal and distal portions of both ECR brevis and longus were also documented by Livingston et al. (2001) by using magnetic resonance imaging techniques before and after dynamic wrist extension and radial deviations. The existence of anatomical partitions was also discussed for what concerns the ECU, muscle with a complex fiber architecture and innervated by a number of nerve branches (Segal et al., 2002). However, our experimental procedure does not provide us with enough data to verify the origin of the inter-subject variability observed in this study. Lastly, although significant according to the ANOVA test, it should be noted that the inter-subject variability influenced the position of the barycenter far less than the task performed (refer to results section for specific statistical values).

### Limitations of the study

It is important to consider that the electrode grid used in this experiment did not cover the whole forearm. The choice of the detection system was limited by the number of channels available in our EMG amplifier. A 128 electrode grid with 1 cm inter-electrode distance was the best compromise in order to cover a large portion of the limb and ensuring an acceptable spatial resolution. In this experiment, we defined the position of the grid to facilitate the detection of the EMG distribution of muscles likely to show activation of subportions (such as ECR, EDC). Grids covering a different area of the skin, with different electrode geometries or with different spatial resolution might result in different levels of statistical significance. This might enhance the localization of some of the EMG sources, especially those of the muscles that in this experiment were close to the edges of the grid, i.e., ECU, brachioradialis, EDC (little) and extensor digiti minimi. In this study, the occurrence of crosstalk might have shifted the position of the target EMG source toward a second source due to the concurrent activation of more than one muscle (subportion). This issue was limited by requiring contractions as selective as possible; in addition, the segmentation process steps were defined in order to limit the influence of accessory muscle activations. In this experiment, the occurrence of crosstalk cannot be completely excluded, especially in those contractions that usually require a certain degree of synergic activation of ore than one muscle (e.g., ECR brevis and longus in wrist extension and radial deviation, Riek et al., 2000). This might result in larger barycenter standard deviations around the mean positions. However, the consistent positions found across subjects, as well as the consistency of both mean positions identified at different force levels, suggest that this effect might have been small with respect to the purpose of this study. A further limitation to this study is that only selective contractions were analyzed. Daily living activities likely involve a co-activation of more than one muscle, as combined finger and wrist movements are required for object grasping and manipulation tasks; multiple EMG sources might result in amplitude distributions less easy to segment, thus worsening the spatial localization of the EMG distributions. Hence, whether this method is valid in conditions different from the ones tested in this experiment should be tested before making assumptions on possible applications of our results.

### Implications

Physiologically, this study confirms that force production in specific directions is associated to the activation of sub-volumes within the dorsal forearm muscles. Most contractions around the wrist and finger joints could be best discriminated along the transverse direction of the forearm; this is in line with the current technology used for commanding upper limb prosthetic devices through surface EMG, as a number of electrodes placed around the forearm circumference is sufficient to discriminate most simple tasks (Farrell and Weir, 2008; Muceli and Farina, 2012). Although results with this electrode configuration are promising, a larger number of degrees of freedom might be controlled if also the longitudinal dimension of the forearm is considered, especially for the pairs of contractions that in this study resulted to be more easily identifiable in the longitudinal direction (e.g., extension vs. radial deviation, middle vs. ring finger extension). Further implications might concern the field of the assessment of upper-limb musculoskeletal disorders. For instance, previous research showed that patients with lateral epicondylalgia have an altered pattern of forearm muscle activation (Coombes et al., 2009); specifically, a significantly lower EMG amplitude was found in the ECR muscle of patients compared to healthy controls (Alizadehkhaiyat et al., 2007; Rojas et al., 2007). As it is known that tendon degeneration mainly occurs in one of the two heads of the muscle (ECR brevis, Nirschl and Pettrone, 1979), it might be worthy investigating whether a spatial distribution of EMG amplitude indicative of a neuromuscular dysfunction predominantly involving the ECR brevis can be found in individuals with lateral epicondylalgia. This might help to define which exercises are most likely to be effective for these patients, supporting and providing more details to the clinical indications that can be found in the study of Rojas et al., (2007).

## Conclusion

In this study, the spatial distribution of EMG amplitude over the proximal, dorsal portion of the forearm was quantitatively described during selected wrist and finger isometric contractions. The position of the EMG sources is in line with the expected position of the main agonist for each of the contractions tested. The spatial properties of the monopolar EMG amplitude distribution can be used to localize EMG sources associated to selective activation of contiguous muscles (e.g., ECU and EDC), muscle heads (e.g., ECR longus and brevis) and compartments existing within a single muscle (e.g., EDC middle and ring). Heterogeneous muscle activation can be investigated by extracting the spatial properties of the EMG amplitude distribution associated to the activation of sources localized in limited muscle sub-volumes.

### Conflict of interest statement

The authors declare that the research was conducted in the absence of any commercial or financial relationships that could be construed as a potential conflict of interest.
